# Evaluation of a Push-Pull Approach for *Aedes aegypti* (L.) Using a Novel Dispensing System for Spatial Repellents in the Laboratory and in a Semi-Field Environment

**DOI:** 10.1371/journal.pone.0129878

**Published:** 2015-06-26

**Authors:** Ulla Obermayr, Joachim Ruther, Ulrich R. Bernier, Andreas Rose, Martin Geier

**Affiliations:** 1 Biogents AG, Weissenburgstrasse 22, D-93055, Regensburg, Germany; 2 Universitaet Regensburg, Institute of Zoology, Universitaetsstrasse 31, D-93053, Regensburg, Germany; 3 USDA-ARS, Center for Medical, Agricultural and Veterinary Entomology, 1600 SW 23^rd^ Dr., Gainesville, Florida, 32608, United States of America; United States Department of Agriculture, Beltsville Agricultural Research Center, UNITED STATES

## Abstract

The increase in insecticide resistant mosquito populations necessitates the exploration of novel vector control intervention measures. Push-pull strategies for insect control have been successful when used in integrated crop pest management. Through the combinatory use of deterring and attracting stimuli, the abundance of insect pests can be changed in a given area. A push-pull strategy might also significantly reduce human-vector contacts and augment existing mosquito control strategies, e.g. through the combination of an attractive trapping system and a potent spatial repellent. Our approach includes the BG-Sentinel (BGS) trap in combination with catnip oil (*Nepeta cataria*), a known spatial repellent for *Aedes aegypti*. To impart a deterrent effect on mosquitoes at a distance, a homogenous and continuous dispersal of volatile repellent compounds is crucial. We have developed a repellent dispensing system that is easy to use and provides a homogenous dispersal of repellent in an air curtain. The use of five 9 V fans and custom-made repellent sachets containing 10% catnip essential oil created a repellent loaded air curtain that provided coverage of an area of 2 m^2^ (1.2 x 1.65 m). Air was sampled at four different heights in the curtain and analysed via thermal desorption (TD) and consecutive gas chromatography—mass spectrometry (GC-MS). Nepetalactone, the main constituent of the oil, was detected in air at a concentration range of 80 to 100 μg/m^3^ and the amounts were comparable at all four sampling positions. When a human volunteer was sitting behind the repellent curtain and a BGS trap was installed in front of the curtain in laboratory push-pull trials, *Ae*. *aegypti* landing collections decreased significantly by 50% compared to repellent-free controls. However, in a semi-field environment, comparable protective effects could not be achieved and further research on suitable repellent concentrations for outdoor implementation will be required.

## Introduction

Vector-borne viral diseases like dengue, dengue hemmorrhagic fever and chikungunya present a major threat to human health. They are transmitted by *Aedes aegypti* (L.) and *Ae*. *albopictus* (Skuse), two widely distributed and very competent vector mosquitoes that mate, feed and oviposit close to human dwellings. Breaking the transmission cycle depends primarily on eliminating or reducing the vector population; however, control measures are often ineffectively applied [1]. Intervention strategies mostly rely on the use of insecticides [2], but traditional methods such as adulticidal fogging can be inadequate to target *Aedes* mosquitoes, as they tend to rest in secluded sites [3]. Indiscriminate or inefficient insecticide application also has led to an increased development of insecticide resistance [4; 5; 6]. A recent study on the susceptibility status of eight *Ae*. *albopictus* populations collected in the United States revealed DDT resistance in 3 strains [7]. According to the authors, continuous monitoring of the insecticide resistance status is absolutely essential since “underlying DDT resistance often results in pyrethroid resistance“. The same group emphasized the serious threat of insecticide resistance to dengue vector control programs in Southeast Asia, South America and the Caribbean where high levels of resistance have been reported [8]. Insecticide resistance in wild mosquito populations necessitates exploration of novel intervention measures. Current vector control measures need to be augmented or replaced by alternative strategies to contend with the growing numbers of resistant populations. A successful strategy used in integrated crop pest management is *push-pull* [9; 10]. Through the combinatory use of deterring and attracting stimuli, the abundance of an insect pest can be changed in a given area by interfering with the ability of the target pest to locate a resource (“push“) and luring it to an alternative source where it is trapped and removed (“pull“). In mosquito control, push-pull strategies have generated great interest over the past few years as they may provide useful techniques to help improve existing control measures. Push components, such as spatial repellents, are used to keep mosquitoes away from human dwellings and trapping systems baited with attractant lures can be used to remove mosquitoes from the intervention area. A recent study from Kenya provides evidence that such a strategy could help to reduce human-mosquito contact [11].

A spatial repellent is defined as a chemical that deters mosquitoes at a distance and inhibits their ability to locate a host [12; 13]. The term has also been used to describe the action of vaporized insecticides that cause knock-down, mortality, or inhibition of feeding. Sublethal doses of highly volatile pyrethroids, like metofluthrin, transfluthrin or allethrin have therefore been suggested to be implemented as spatial repellents in push-pull control strategies [14; 15; 16]. Over the past few years there has also been an increased effort to investigate plant-derived, non-insecticidal spatial repellent candidates and few have been identified, like linalool and dehydrolinalool [17], geraniol [18] and catnip oil (*Nepeta cataria)* [19], the most promising one. The major constituent of catnip oil, nepetalactone, is repellent to planthoppers, ants, caddisflies, beetles [20], cockroackes [21], mosquitoes [22] and stable flies [23]. Several laboratory studies have indicated spatial effects of catnip against *Ae*. *aegpyti*. Catnip was more effective than Deet in inhibiting *Ae*. *aegypti* attraction to human odors in triple-cage olfactometers [24], reduced mosquito attraction to a human finger by more than 70% in y-tube olfactometer assays [25], caused 60% of a test mosquito population to fly from a repellent treated chamber to a repellent-free chamber in trials without human odors [26] and elicited an 80% escape rate in contact trials within excitorepellency test chambers [27].

The host finding process of *Ae*. *aegypti* has been extensively studied and is well understood. Several compounds naturally found on human skin play an important role for mosquito-host attraction, like L-lactic acid [28; 29], ammonia [30], fatty acids [31], acetone and dimethylsulfide [32]. In combination with traps, synthetic kairomone blends can help to increase catching efficacy or enhance target species selectivity [33]. The BG lure (Biogents AG, Regensburg), a commercially available kairomone dispenser, has been especially designed for *Aedes (Stegomyia)* species, combining three synthetic compounds that are highly attractive to *Ae*. *aegpyti* and *Ae*. *albopictus*: L-lactic acid, hexanoic acid and ammonia. The BG lure is commonly used in combination with the BG-Sentinel (BGS) trap, currently the most successful trapping tool to target *Aedes (Stegomyia)* species [34; 35]. Even in the absence of CO_2_, BGS traps equipped with a BG lure dispenser caught significantly more *Ae*. *aegypti* than CO_2_-baited EVS traps [36] and significantly more female *Ae*. *albopictus* than CO_2_-baited CDC traps [37]. Based on its superiority in capturing *Aedes (Stegomyia)* species compared to other standard trapping systems, the BGS has been suggested to serve as a pull component in *Ae*. *aegypti* push pull control strategies [14; 15; 25].

In contrast to our extensive knowledge on mosquito host attraction and trapping technologies, finding a powerful spatial repellent that is nonhazardous, long lasting and releases an unobstrusive odor is by far the greater challenge. Some groups investigated the potential of low-dose insecticides to serve as spatial repellents [38; 39] while others focussed on identifying non-toxic plant-derived compounds that reduce mosquito host attraction [17; 18; 40].

In a recent study, our group presented a simple repellent dispensing device for the indoor evaluation of candidate spatial repellents to be used in push-pull systems [25]. The device consisted of a perforated polyethylene (PE) tube and compressed air connection. Repellent formulations were released through fine holes in the PE tube, creating a repellent loaded air curtain. Test mosquitoes had to fly through this curtain to reach a BGS trap that served as the attracting stimulus. In these tests, the most successful materials, catnip oil (*Nepeta cataria*) and a mix of catnip oil and homopiperazine, reduced trap catches by 50% to 90%. However, the system had its drawbacks: the created air curtain was heterogenous and contained gaps that allowed easy access to the mosquitoes leading to a great variation in the obtained results. A homogenous repellent dispersal was determined to be crucial for the success of a push-pull system and there was a need for a more reliable device that could easily be implemented within more realistic settings. In the present study, we developed and compared two new experimental set-ups for repellent dispersion, the “shower head-”(SHS) and “five fan system”(FFS). Results obtained with the new systems were compared to determine which of the two set-ups provides a more homogenous repellent-loaded air curtain. In semi-field tests, the efficacy and the applicability of the system were investigated under more realistic outdoor conditions.

## Material and Methods

### Chemicals

Pure catnip (*Nepeta cataria*) essential oil was purchased from Aromaland (Röttingen, Germany). For SHS and olfactometer trials, catnip oil was diluted with ethanol (96%, p.a.) to final concentrations of 2.5% and 10% (w:w). For trials using the FFS, catnip oil was diluted in paraffin oil to a final concentration of 10% (w:w). Menthalactone (≥ 99% purity) used as internal standard for the chemical analysis was obtained from Sigma-Aldrich (Taufkirchen, Germany).

### Repellent Sachets

FFS trials utilized repellent sachets to disperse volatile active ingredients. Each sachet consisted of a 7.5 x 100 cm piece of Stericlin tube (Vereinigte Papierwarenfabrik GmbH, Feuchtwangen, Germany) filled with 100 g polymer Ingeo 4043D granules (NatureWorks LLC, Minnesota, USA) that had been loaded with either 10 g of a 10% catnip in paraffin formulation or 10 g of paraffin only (controls). One surface of the Stericlintube is a non-permeable, transparent foil while the other consists of a Tyvek membrane that is permeable for gases but not for liquids.

During trials, dispensers were hung above the fans inside the FFS. In the laboratory, one trial lasted up to 15 min, in the semi-field dispensers were operated for 1 h. In between trials, dispensers were coiled and stored at room temperature inside a hermetically sealed PE box (14 x 10 x 7 cm). The loss of volatile ingredients from the sachets was measured gravimetrically after each experiment. Paraffin dispensers remained at the same mass in laboratory trials, but in the semi-field the loss reached an average of 0.009 g/h. Based on these findings, the mass loss in repellent dispensers was attributed to the evaporation of catnip oil from the polymer granules. In laboratory trials, catnip oil evaporated at approximately 0.06 g/h; in the semi-field the weight of catnip dispensers decreased by approximately 0.05 g/h. Repellent dispensers contained an initial amount of 1 g catnip essential oil (dissolved in 9 g paraffin); they were replaced as soon as their mass had declined by 0.5 g to ensure that sufficient amounts of active volatiles were still present in the dispenser. In the laboratory, catnip sachets could be used for at least 8 h, in the semi-field situation dispensers lasted for an average of 10 h.

### Test Mosquitoes

Six to 20-d-old *Ae*. *aegypti* females were used for all laboratory and semi-field tests. Preliminary behavioral assays in y-tube olfactometers with our lab colony demonstrated comparable susceptibility for spatial repellents at 6–20 days after emergence while responses showed greater variability when mosquitoes were at a younger age (1–5 d). The colony was originally obtained from BAYER AG (Monheim, Germany) in 1998 and has been maintained in our facilities over the past 17 years.

Mosquitoes were reared at 26 ± 1°C and 70 ± 5% relative humidity (RH) under a photoperiod of 12:12 (L:D) h for the laboratory trials. After hatching of the eggs, larvae were kept in a water basin (30 x 30 x 10 cm) filled with a 1:1 mixture of tap water and deionized water and fed with Tetramin fish food flakes (Tetra GmbH, Melle, Germany). Pupae were transferred into breeding cages (40 x 40 x 20 cm). Adult mosquitoes were provided with a 10% glucose solution on filter paper.

Adult mosquitoes used in all experiments were selected based on host seeking behavior. The breeding cage contained a circular opening covered by fine mosquito netting in the left wall, while the right wall was fitted with a port and rotating door, where a transfer container could be attached. The transfer container consisted of a perspex cylinder with rotating door on one end and a cover made from fine mosquito netting at the other end. A fan running at 7.5 V was connected to the opening in the left wall of the breeding cage, while a human hand was held against the transfer container on the opposite side of the cage and rotating doors were opened. Female mosquitoes seeking a bloodmeal flew upwind into the transfer container because they were attracted to the skin odors.

In semi-field tests, *Ae*. *aegypti* females from the Orlando strain were used. Previous olfactometer trials verified the positive spatial repellent activity of catnip oil against this strain [24]. The colony has been maintained since 1952 at the facilities of the United States Department of Agriculture, Agricultural Research Service, Center for Medical, Agricultural and Veterinary Entomology (USDA-ARS-CMAVE) in Gainesville, Florida, following a similar protocol. In the morning of each test day, host-seeking mosquitoes were lured out of the breeding cages into a collection trap by natural host stimuli and then immobilized at 4°C for 30 min. Mosquitoes were counted into batches of 100 females on a cooled tray, placed into holding containers, provided with 10% sugar water and kept at 26 ± 1°C and 70 ± 5% until the start of the tests. A maximum of 6 tests were performed per day. In order to be able to distinguish mosquitoes from different rounds they were labeled with different luminous powders (BioQuip Products Co., Gardena, CA, USA) inside their holding containers.

### Room Tests with Repellent Dispensing Systems and BGS Trap

Tests followed the procedure described previously [22]. Tests were performed in an air-conditioned 40.25 m^3^ windowless room (4.6 x 3.5 x 2.5 m) with artificial light from two fluorescent tubes (350 Lux). The temperature and humidity of the air in the room were set to 25 ± 1°C and 60 ± 5% RH, respectively. Results from previous olfactometer trials at 28 ± 1°C and 80 ± 5% RH indicated that the spatial activity of catnip was not impacted at this higher temperature and humidity combination (unpublished data). Clean, warm and humid air entered the room through an opening in the ceiling and exited the room through a second opening 4.5 m distant on the far side. A tent structure comprised of cotton fabric was built around the air entry with bottom edges held on the floor by wooden bars. The tent measured 1.2 x 1.2 x 2.5 m (L x W x H) and had three closed sides and an open entrance (1.2 x 1.8 m) on the forth side. The repellent dispensing systems were installed at the top of the tent entrance.

### Experiment 1—Shower head system (SHS)

The SHS consisted of three conventional shower heads (Mixomat LED Handbrause, BAHAG AG, Mannheim Germany). Each shower head was connected to a poly propylene (PP) container (12 x 12 x 8 cm) by PE tubing (S1 Fig). Containers included a second opening for the introduction of pressurized air. For each test, three round filter papers (Schleicher & Schuell BioScience GmbH, Dassel, Germany) were treated with 500 μl of the 10% ethanolic catnip formulation and enclosed in the PP containers. Pressurized air was passed through the containers to pick up the repellent volatiles evaporating from the filter paper. Smoke experiments (data not shown) indicated a greater density of the curtain between 0.3 and 1.45 m above ground, gaps were noticed between the three shower heads and the curtain appeared to thin out towards the ground (S2 Fig). The volume of the created air curtain was estimated at approximately 0.18 m³ (1.65 x 1.2 x 0.09 m). The speed of the repellent loaded air that left the shower heads was measured with an anemometer, it reached 0.1–0.2 m/s at a distance of 1 cm from the nozzles. This air flow corresponds to the one measured in previous trials with the PE tube system (Obermayr et al., 2012). In control trials, new shower heads were used and filter papers were treated with ethanol only. In all experiments, air flow was switched on 5 min before test mosquitoes were released into the room.

### Experiment 2—Five fans system (FFS)

**Fig 1:**
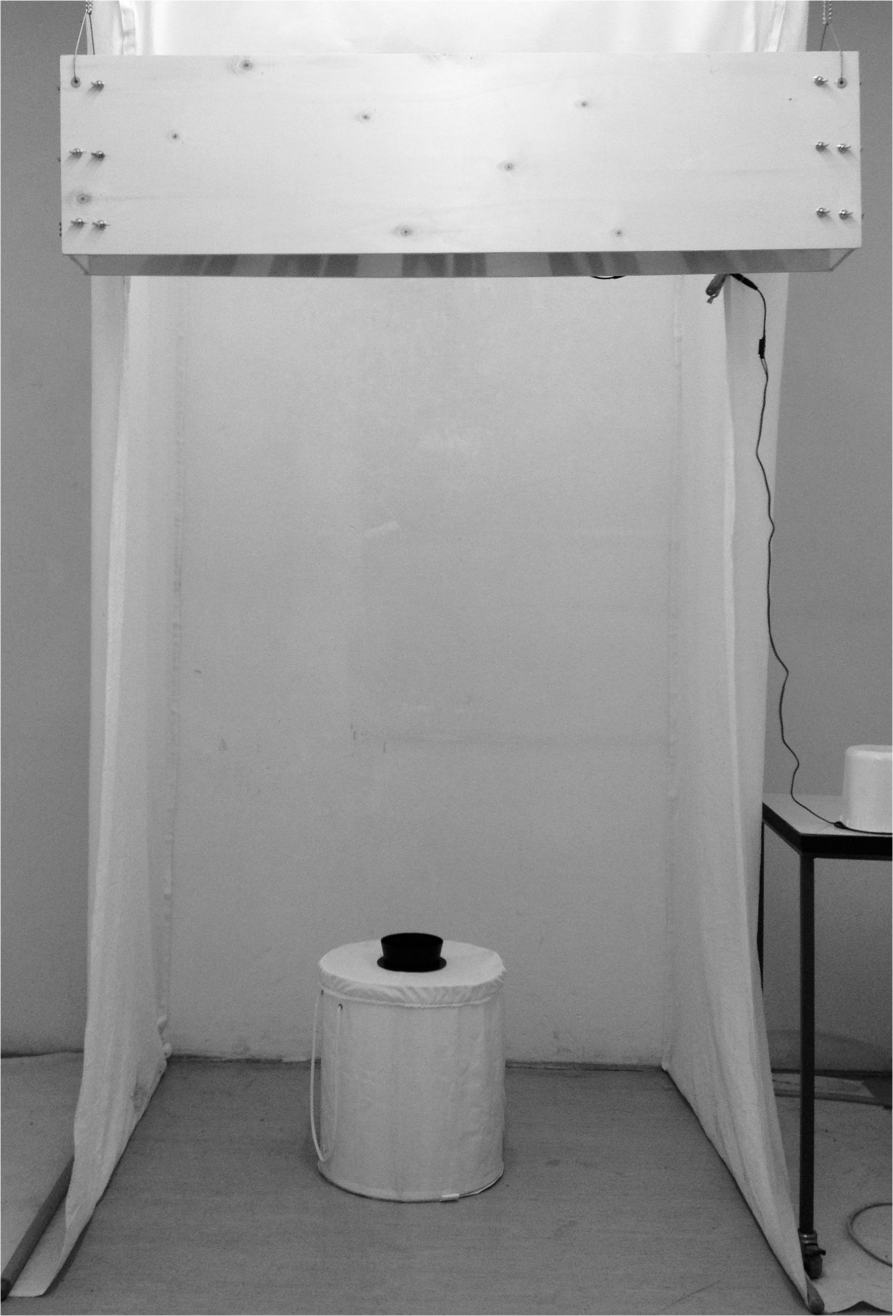
Laboratory test set-up showing the tent structure, BGS trap and FFS (front view).

The FFS consisted of a 120 x 15 x 30 cm wooden frame into which five 12 V DC fans were mounted equidistantly with the down flow facing the tent opening (Fig 1). The fans could be operated at 3, 4.5, 6, 7.5, 9 and 12 V, with each voltage creating different air speeds in the tent opening (S1 Table). Control tests were used to identify the speed that did not generate a mechanical barrier to the mosquitoes. Mosquitoes were able to overcome the air curtain at all tested speeds (S3 Fig), even at 0.8–2.0 m/s which were measured in the center of the opening when fans were operated at 12 V. The lowest variation was found at 9 V, when wind speeds in the center of the opening reached 0.8–1.4 m/s. Based on these findings, all consecutive trials were conducted with fans operating at 9 V. Smoke experiments (data not shown) indicated that the entire tent opening was uniformly covered (S4 Fig), the volume of the generated air curtain was estimated at approximately 0.24 m³ (1.7 x 1.2 x 0.12 m)

**Fig 2:**
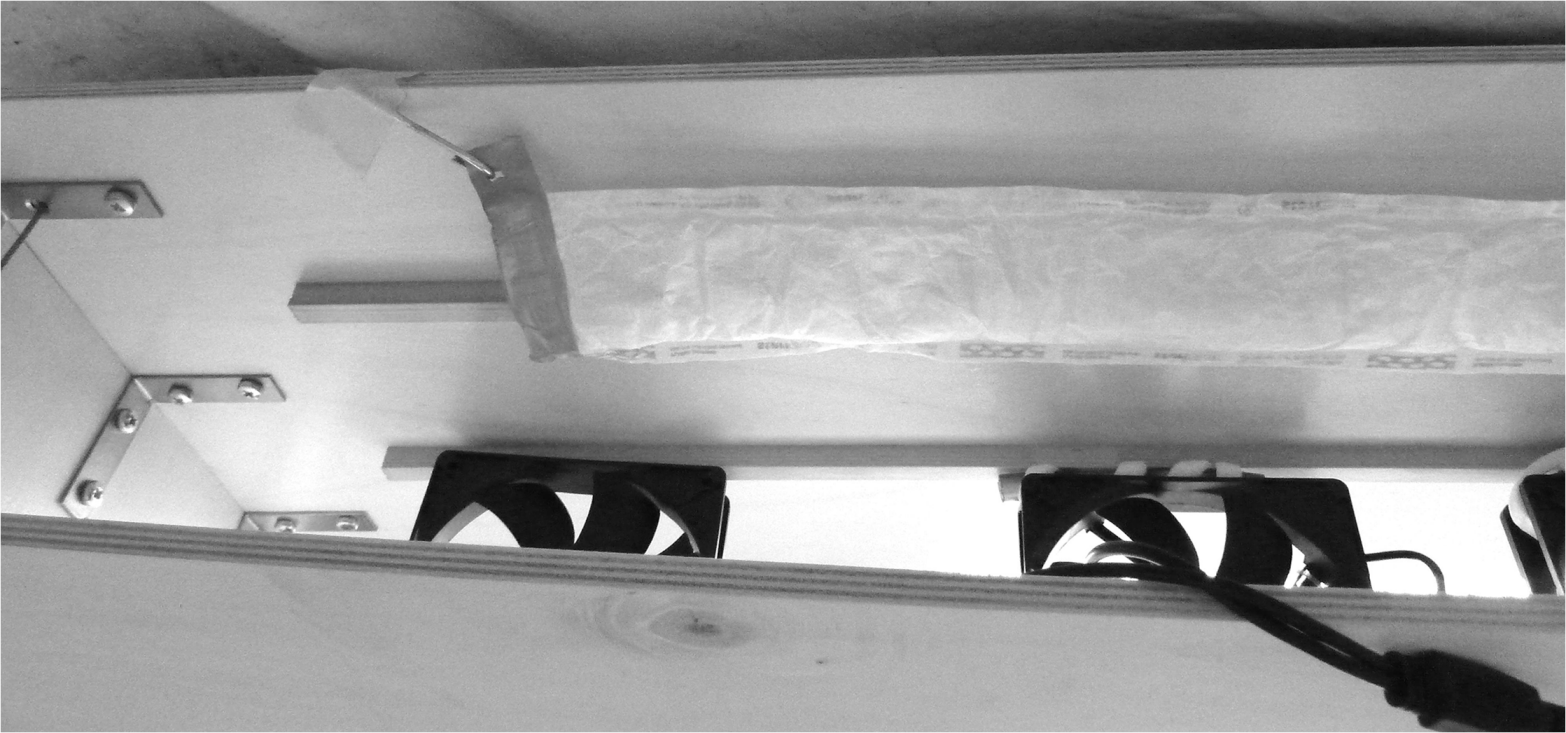
Stericlin repellent sachet attached to the FFS with the permeable side facing the fans.

Prior to the start of a test, a repellent sachet was hung into the frame with its permeable Tyvek-side facing the row of fans (Fig 2). Mosquitoes were released immediately after the system was switched on. In all experiments, a BGS trap fitted with a BG-Lure dispenser was used as a proxy for a human target and placed inside the tent to attract host-seeking *Ae*. *aegypti*. Those mosquitoes that passed the air curtain were captured by the trap. For each individual test, 10 mosquitoes were released into the room at the side furthest away from the tent and allowed to respond for 15 min. Preliminary room tests had revealed that this was the maximum time period needed for all mosquitoes to be caught by the BGS trap and/or volunteer At the end of the test time, the investigator entered the room and documented the trap catch rate. Still free flying mosquitoes were aspirated with a modified hand-held vaccuum cleaner. Mosquitoes that did not approach the investigator or that were still sitting inside the transport cage were recorded as inactive. For each dispensing system, 10 replicates were conducted per treatment (repellent and control). Treatments were tested in a randomized order. To avoid an accumulation of the volatile stimuli, the room was aerated for 30 min before the next test was conducted.

### Laboratory Push Pull Set-Ups

In experiment 3, two BGS traps were used with the FFS to simulate a push-pull situation. One trap served as an attracting stimulus inside the tent (BGS I) while the second one was used as an alternative target on the outside (BGS O). Capture rates of BGS I and BGS O were measured in the presence of catnip and compared to control trials with paraffin.

In experiment 4, BGS I was replaced by a human bait to determine if the combinatory effects of a BGS trap plus repellent curtain can decrease human landing rates. Human landing collections were performed in [a] absence of BGS O and absence of repellent, [b] presence of BGS O and absence of repellent, [c] absence of BGS O and presence of repellent and [d] presence of BGS O and presence of repellent (= push-pull situation). In both experiments, a total of 10 replicates were performed per set-up.

### Quantification of Nepetalactone

Nepetalactone is the main component of catnip oil [41] and constituted 84% of the sample used in our experiments (data not shown). Therefore, we quantified the concentration of this compound in the air curtain of the SHS and the FFS set-ups and for comparison also in the Y-tube olfactometer used in our previous study, in which catnip oil was found to be highly efficient against *Ae*. *aegypti* [25]. For quantification, we performed headspace analyses by thermal desorption gas chromatography coupled to mass spectrometry (TD-GC-MS). Volatile sampling was performed by aspiration of the volatile laden air for 30 s at a flow rate of 200 ml/min through pre-packed thermal desorption filters filled with a combined Tenax-TA/Carboxen adsorbent (Sigma-Aldrich, Taufkirchen, Germany). After volatile sampling, 5 ng of the internal standard menthalactone (dissolved in 2 μl methanol) were applied to each adsorbent tube and the solvent was removed by purging the filter for 5 min in a stream of nitrogen at a flow rate of 60 ml/min. Filters were thermally desorbed for 8 min at 250°C using an automated Shimadzu TD20 thermal desorption system. The desorber was connected to a Shimadzu 2010 plus GC/MS system (Shimadzu, Duisburg, Germany) equipped with a non-polar BPX-5 column (30 m length, 0.32 mm i.d., 0.25 μm film thickness, SGE Analytical Science, Milton Keynes, UK). Helium was used as carrier gas at a linear velocity of 50 cm/s. The GC program started at 40°C and was ramped at a rate of 3°C/min to 163°C and then at 10°C/min to 280°C (final hold 6 min). The MS was operated in electron impact (EI) mode at 70 eV and a scan range from 35–600/mz. A calibration curve was generated by applying 1 μl aliquots of catnip oil dilutions in methanol representing 0.84–168 ng nepetalactone and 5 ng of the internal standard to the adsorption tubes. The solvent was removed as described above and before the standard samples were analyzed with the same TD-GC-MS method.

Air samples were collected at four different heights (position 1 = 137 cm (above ground); position 2 = 107 cm; position 3 = 77 cm and position 4 = 44 cm) and at four different points in time after switching on the SH or FF dispensing systems (at 0, 5, 10 and 15 min). Prior to volatile collection in the y-tube olfactometer, 30 μl of a 2.5% ethanolic catnip solution were dropped onto filter papers and held into the air stream of the apparatus after the solvent had evaporated (30 s). This treatment has been shown to be highly efficient in repelling *Ae*. *aegypti* (for details see [25]). Volatile sampling (n = 10 replicates) was done as described above whereby the sample tube was positioned at the bottom center of the base leg.

### Semi Field Test

Semi field experiments of the FFS were conducted at the USDA in Gainesville, Florida, between September 18 and 30, 2014. All experiments were performed inside a large outdoor cage (9.1 m wide x 18.3 m long x 4.9 m high, gabled to 6.1 m) covered with mosquito screen to allow entry of precipitation and wind. The cage contained vegetation and was equipped with a 12–14 personnel tent (HDT Base-X Model 305 Shelter, HDT Global, Solon, Ohio, USA; 5.5 m wide x 7.6 m long x 2.5 m high). The FFS set-up was installed at the top of a tent opening (2 m high x 1.3 m wide). All mosquitoes used in semi field tests came from the USDA colony (see above).

**Fig 3:**
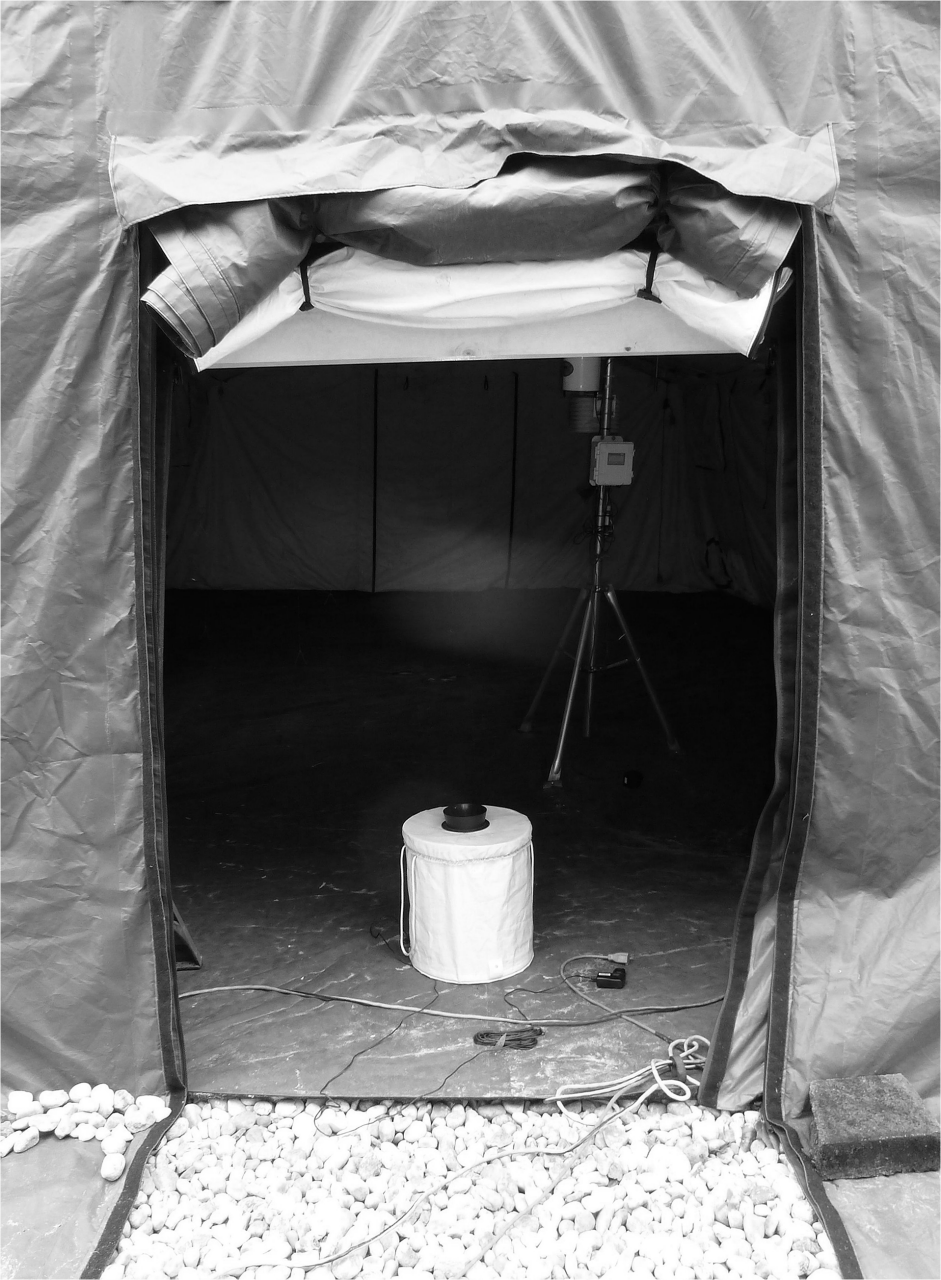
Semi-field set-up showing the tent inside the outdoor cage, BGS trap and FFS.

Due to the limited time outdoor test facilities were available, two experimental set-ups were used in semi field trials: [1] A BGS trap fitted with a BG lure dispenser was installed inside the tent as an attracting stimulus (Fig 3). Trap catch rates were documented in the presence of catnip and compared to control trials with paraffin oil only. [2] A human volunteer sat inside the tent to attract mosquitoes to fly through the air curtain while one BGS trap was installed outside (= push-pull set-up). Compared to laboratory trials, the greater space provided by the semi-field set-up necessitated a longer testing period. Mosquitoes were released at the far end of the cage and allowed for 1 h to respond to the test stimuli, following the USDA standard testing procedure for semi-field trials involving traps (Daniel L. Kline, personal communication). Mosquitoes approaching the volunteer were aspirated into collection tubes attached to a modified hand-held vacuum cleaner. At the end of a test, BGS catch bags and collection tubes were removed and stored at -20° for later counting. A total of 10 replicates were performed per treatment (repellent and control).

### Ethics Statement

The volunteer in this study provided written informed consent to conduct human landing accounts as described in the section on "volunteering for the semi-field test," which is part of the Instituational Review Board (IRB) Study #636–2005, approved by the University of Florida IRB-01.

### Data Analysis

For both, laboratory and semi field tests, mean percentages and corresponding standard deviations of mosquitoes caught by the BGS trap and volunteer were calculated from catnip and control trials. Mean percentages were subjected to an arcsine transformation prior to the statistical analysis. Means from laboratory experiments 1, 2, 3, and semi field were compared independently by non-parametric Mann-Whitney-U-test. Mean human landing collections from experiment 4 were compared using Kruskal-Wallis followed by pairwise Mann-Whitney-U-test with Bonferroni corrected p-values to look for significant differences between the four test scenarios.

For nepetalactone quantification, mean quantities and corresponding standard deviations of each sampling position and point in time were calculated. Mean quantities were compared using two-way analysis of variance (ANOVA) with Tukey´s honest significant difference (HSD) test as a post hoc test in order to examine if position and time had an influence on nepetalactone quantities. A p value ≤ 0.05 was regarded as statistically significant. All statistical tests were performed using PAST version 3.04 [42].

## Results

### Room tests of the SHS and FFS

**Fig 4:**
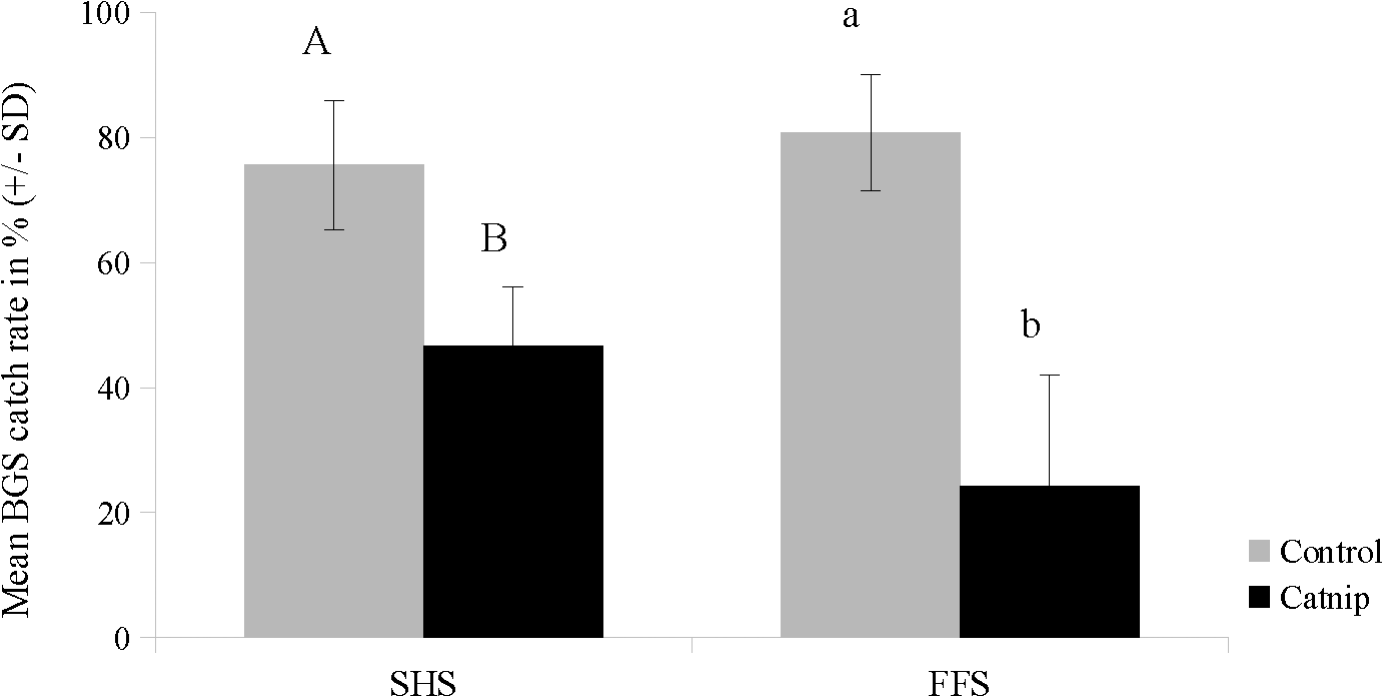
BGS catch rates (means ± standard deviation) of *Ae*. *aegypti* in control (grey) and catnip (black) trials of the SHS (experiment 1) and FFS (experiment 2). Different letters indicate significant differences in mean BGS catch rates in tests of the SHS (uppercase) at p = 0.0047 (Mann-Whitney-U-test, n = 10) or FFS (lowercase) at p = 0.00018 (Mann-Whitney-U-test, n = 10).

The first experiment evaluated the new SHS set-up. Compared to control trials, BGS capture rates were reduced significantly in the presence of catnip (U = 0.00; Z = -2.8271; P = 0.0047) (Fig 4). When catnip was dispensed, the average BGS catch rates dropped by 38%. Experiment 2 involved the FFS in combination with Stericlin sachets and BGS trap. In the presence of catnip, BGS catch rates were reduced significantly by 70% (U = 0.00; Z = -3.7489; P = 0.00018) (Fig 4).

**Fig 5:**
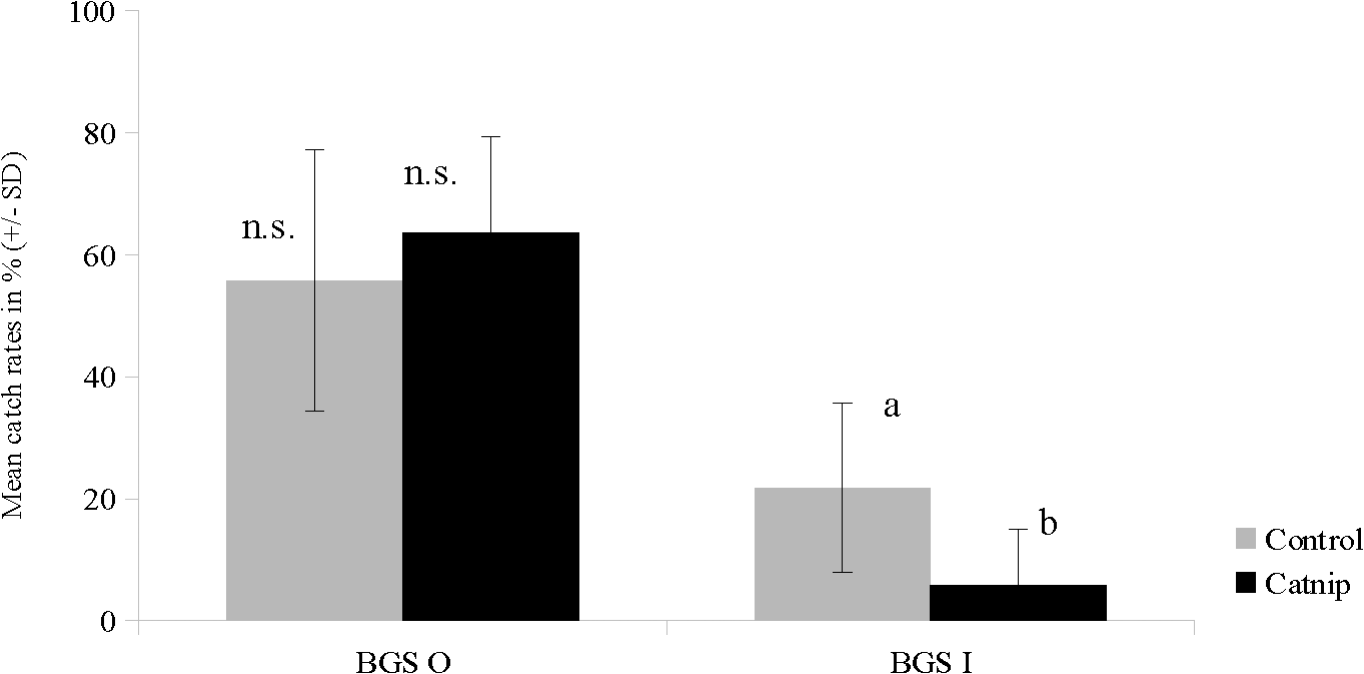
*Ae. aegypti* recapture rates (means ± standard deviation) of BGS O and BGS I in control (grey) and catnip trials (black) of the FFS. Different lowercase letters indicate significant differences at p = 0.017 (Mann-Whitney-U-test, n = 10). n.s. = non-significant.

A simple push-pull set-up using the FFS with two BGS traps was tested in experiment 3 (Fig 5). In the presence of catnip, BGS I catch rates significantly decreased by > 70% (U = 19; Z = -2.3916; P = 0.0167) whereas mean BGS O catches did not significantly differ from control trials (U = 39; Z = -0.7988; P = 0.4243).

**Fig 6:**
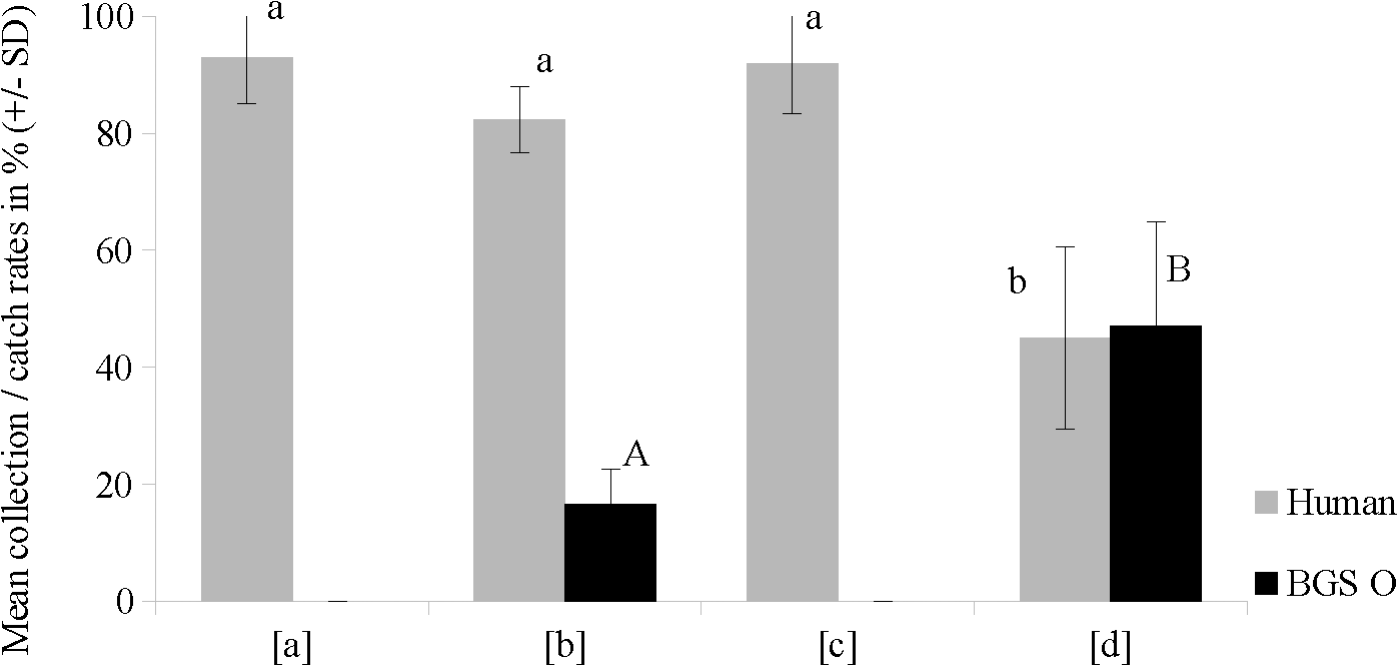
Human collection rates (black) and BGS catch rates (grey) (means ± standard deviation) of *Ae*. *aegypti* in laboratory room tests of the FFS in combinaton with Stericlin dispensers for catnip oil dispersal (experiment 4). Test scenarios: [a] no repellent, BGS O absent; [b] no repellent, BGS O present; [c] repellent, BGS O absent and [d] repellent, BGS O present (= push-pull situation). Different letters indicate significant differences in mean BGS trap catch rates (uppercase) at p < 0.0004 (Mann-Whitney-U-test, n = 10) or mean human landing rates (lowercase) at p < 0.0009 (Mann-Whitney-U-test, n = 10).

BGS I was replaced by a human volunteer in experiment 4, representing a more realistic push-pull set-up (Fig 6). Human landing collections showed significant differences between the 4 scenarios (H = 25.79; p = 6.28E-06): in test scenarios [a], [b] and [c] the majority of the released mosquitoes reached and landed on the volunteer, resulting in human landing collections of 92.9 ± 7.9%. When catnip was used in combination with BGS O in test scenario [d], human landing collections decreased significantly by 45% to 50% compared to test scenarios [a] (P = 0.0009), [b] (P = 0.0008) and [c] (P = 0.0009) while BGS trap catches significantly increased (U = 3.5; Z = -3.5418; P = 0.00039).

### Quantification of nepetalactone

Volatile collections with the SHS revealed that both position of the adsorption tubes (F = 19.3; df = 3; P = 2.28E-10) and sampling time (F = 3.088; df = 3; P = 0.029) significantly influenced nepetalactone quantities in the air curtain (Table 1). There was also a significant interaction between positions and point in time (F = 2.045; df = 9; P = 0.039). Mean nepetalactone quantities fluctuated over time, at positions 1 and 4 greater quantities were found after 15 min compared to 0 min. At 5, 10 and 15 min mean quantities collected at position 1 were greater than the ones obtained at positions 2, 3, and 4.

Compared to the SHS, quantification data of the FFS indicated a more homogenous and constant nepetalactone dispersal (Table 1). Mean nepetalactone quantities were not significantly different between the four positions (F = 1.336; df = 3; P = 0.27) and quantities did not significantly change over time (F = 1,84; df = 3; P = 0.1493). A significant interaction between the positions and point in time was not detected (F = 0.522; df = 9; P = 0.8531).

Nepetalactone quantities were greater in samples taken from the FFS: mean quantities collected from all positions at 0 min (81.4 ± 37.5 μg/m^3^) were significantly greater than mean olfactometer collections (54.9 ± 13.3 μg/m^3^) (U = 43; Z = -2.363; P = 0.0181). Overall, mean nepetalactone quantities (pooled from all positions and every point in time) were significantly greater in FFS collections (91.0 ± 26.3 μg/m^3^) compared to collections from the SHS (57.9 ± 65.0 μg/m^3^) (U = 2237; Z = -7.3949; P = 1.41E-13). At sampling point 0 min, mean nepetalactone quantities of the SHS (40.0 ± 77.9 μg/m^3^) were also significantly lower than mean olfactometer collections (U = 57; Z = -3,2079; P = 1.34E-03).

**Table 1:** Concentration of nepetalactone (mean ± SD) as determined in the air curtains of two dispensing systems and y-tube olfactometer by thermal desorption headspace gas chromatography-mass spectrometry (TD-GC-MS).

Position	Mean nepetalactone quantities [μg/m^3^] ± SD			
	SH dispensing system (n = 9)			
	0 min	5 min	10 min	15 min
1	48.3 ± 25.4 a	108.3 ± 56.7 ab/A	135.5 ± 57.8 b/A	164.3 ± 89.9 b/A
2	21.5 ± 15.2	22.4 ± 14.6 B	24.9 ± 11.6 B	28.7 ± 17.2 B
3	62.1 ± 14.3	33.1 ± 16.1 B	42.9 ± 20.6 B	45.1 ± 17.1 B
4	25.2 ± 27.1 a	49.0 ± 30.1 ab/B	44.5 ± 18.8 ab/B	68.0 ± 29.9 b/B
	FF dispensing system (n = 5)			
1	64.4 ± 10.9	88.9 ± 18.4	96.6 ± 10.4	89.2 ± 20.3
2	94.0 ± 41.9	98.1 ± 11.4	110.4 ± 24.3	110.1 ± 8.1
3	97.4 ± 41.8	80.4 ± 13.1	97.2 ± 29.6	101.8 ± 21.5
4	66. ± 26.8	83.1 ± 19.7	94.7 ± 16.0	82.2 ± 16.0
	Y-tube olfactometer (n = 10)			
	54.9 ± 12.6	-	-	-

Nepetalactone quantites are given in relation to the distance of the sampling point and the time passed after the start of the experiment. Position 1: 137 cm above ground, position 2: 107 cm, postion 3: 77 cm and position 4: 44 cm. Different lowercase letters indicate significant differences between rows for each set-up at p < 0.03 (Tukey´s HSD-test, n = 9), capital letters indicate significant differences inside columns at p < 0.007 (Tukey´s HSD-test, n = 9).

### Semi field trials with the FFS set-up

**Fig 7:**
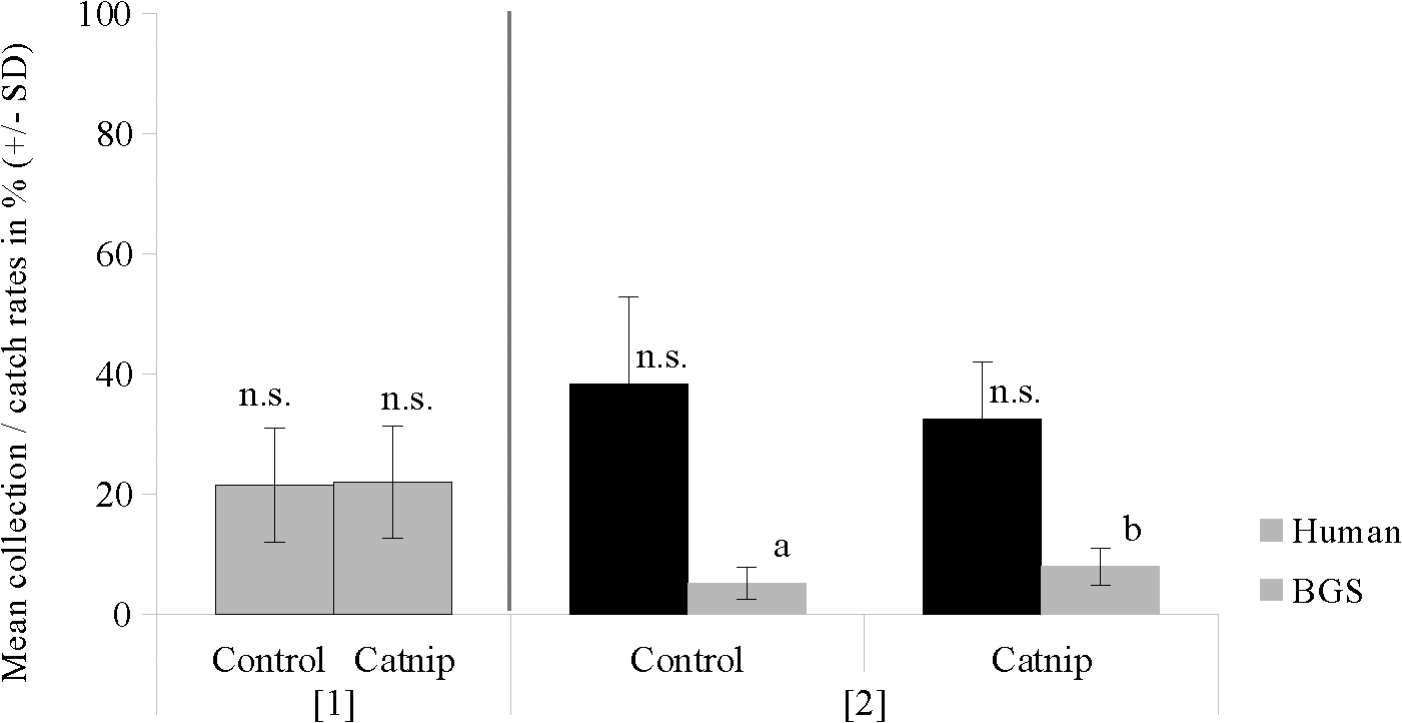
Human collection & BGS catch rates (means ± standard deviation) of *Ae. aegypti* in semi-field trials of the FFS. The x-axis shows test scenarios: [1] BGS as attracting stimulus inside the tent and [2] Human volunteer inside the tent plus BGS O (= push-pull situation). Different lowercase letters indicate significant differences at p < 0.04 (Mann-Whitney-U-test, n = 10). n.s. = non significant.

When the BGS trap was used as attracting stimulus in set-up [1], recapture rates in control and catnip trials were not significantly different (U = 45; Z = -0.3402; P = 0.7337) (Fig 7).

In trials involving the push-pull set-up [2] human landing collections were slightly reduced by 15% in the presence of catnip but showed no significant differences to control trials (U = 39; Z = -0.7943; P = 0.427). BGS trap catch rates increased by 30% in the presence of catnip and were significantly higher compared to control trials (U = 22; Z = -2.082; P = 0.037) (Fig 7).

## Discussion

The establishment of push-pull strategies in mosquito control is a subject of great interest but the successful implementation of such a strategy has not been reported yet. One critical aspect is finding the proper spatial repellent and an effective means of dispensing it. With the FFS, we have developed an application-oriented, easy-to-use spatial repellent dispensing system that facilitates a homogenous dispersal of repellent, a crucial parameter for the successful implementation of push-pull strategies. Indoor volatile collections from the FFS and consecutive nepetalactone quantification via TD-GC-MS showed constant and comparable amounts of active ingredient at each of the four sampling positions and throughout the entire sampling period. Smoke experiments used to visualize the air movement inside the curtain supported the assumption of a homogenous dispersal within the FFS. Further research on the SHS was discontinued, as both smoke experiments and nepetalactone quantification indicated gaps or areas of lower repellent density within the air curtain that most likely provided easy access to the mosquitoes. In addition, the dependence on pressurized air also impeded the overall applicability of this technology in an outdoor setting. The FFS is an easy to use alternative, however the fans require (battery) power and this could also be an impediment to using this technology in disease endemic settings.

A combination of the FFS and catnip essential oil plus BGS trap provided promising effects in a confined space. Within the laboratory setting, human landing collections were reduced by 50%; however when transferred to a semi-field environment, protective effects were not as distinct. Although the BGS O catch rates increased significantly in the presence of catnip odors, human landing collections were only reduced slightly. Future research needs to investigate if these limitations can be overcome, *e*.*g*. through (1) the use of dispensers that emit greater quantities of the essential oil, (2) the implementation of CO_2_ as additional trapping cue and (3) the use of multiple BGS traps.

When tested in a different semi-field set-up, catnip was reported to also work as a spatial repellent against *An*. *gambiae* [43]. Mosquito Magnet X (MM-X) traps baited with CO_2_ and odor blends were used to follow mosquito house entry. When catnip was dispensed outdoors at the four corners of an experimental hut, indoor trap catches were reduced by 50% but spatial effect were not examined in the presence of a human volunteer. Our work demonstrates that human-vector contact can be reduced by catnip in a confined area but for a successful outdoor implementation of push-pull we still need to extend our knowledge on the characteristics, capacities and limitations of spatial repellents.

The deterrence elicited by a neurotoxic compound can cause mosquitoes to rest and seek shelter, a behavior that was observed in semi-field studies in Tanzania [44]. In allethrin trials, Mosquito Magnet trap catch rates decreased in treated areas compared to insecticide free controls and mosquitoes were found to rest on walls and vegetation inside the experimental area without showing any host-seeking behavior. When *Ae*. *aegypti* mosquitoes were pre-exposed to common insecticides and subsequently introduced into recapture trials with BGS traps, pre-exposure to transfluthrin significantly reduced BGS trap catch rates in trials immediately after exposure [45]. These findings emphasize that the success of a push-pull control strategy strongly depends on the characteristics of the push component; the target mosquito needs to be deterred but still be attracted to an alternative host and such a reaction might be impeded by the neurotoxic action of pyrethroids. Not only for these reasons but also to avoid human exposure to the potential hazards of pyrethroids, there has been an increased effort to discover alternative, non-toxic spatial repellents and some groups suggested to use plant-derived chemicals as a safe alternative to pyrethroids for indoor personal protection [46]. Field data on the impact of plant-derived compounds on human landing rates are scarce and up to now the protective effects of catnip on humans have never been examined under outdoor conditions.

In general, the impact of a spatial repellent seems to be restricted to short distances and minimal air movement [46] and effects are greater in the presence of homogenous „bubbles”compared to a point-source release of active ingredient [39]. With the FFS, we describe here a repellent dispensing system that created a homogenous repellent air curtain in an indoor set-up. In close proximity to a human host, mosquito-host attraction significantly decreased in the presence of a catnip enriched air curtain and a BGS trap. In our laboratory experiments, part of the test mosquitoes were found to hover in front of the repellent curtain, a behavior we defined as “repellent-initiated hesitation“, and eventually some of them got caught by the BGS trap. This hesitation behavior was apparently elicited by nepetalactone concentrations that were sufficient to deter or confuse the test mosquitoes but did not inhibit attraction as some mosquitoes still flew through the curtain while others were attracted to the BGS trap.

When *Ae*. *albopictus* was exposed to 0.013 μg/cm^3^ vapors of geraniol or anisaldehyde, no noticeable changes in their host-seeking ability were observed. After being exposed to higher doses (0.25 μg/cm^3^) of the same compounds, host-seeking ability decreased by 70–80%, indicating a dose-dependent inhibition in the host seeking behavior [47]. Compared to these physiologically critical doses, mosquitoes were exposed to far lower concentrations of nepetalactone (0.08–0.1 ng/cm^3^) in our FFS experiments and within this range, host-seeking was not inhibited but mosquito host finding was slightly delayed. However, such an effect could not be observed when the FFS was evaluated under outdoor conditions. Experiments were conducted in September 2014, at an average ambient temperature of 25.9°C, 77.5% relative humidity and low to light wind speeds ranging between 4 and 16 km/h. Air movement most likely had a great impact on the integrity of the repellent curtain. In contrast to laboratory trials with nearly static conditions, wind could pass through the outdoor cage and thereby dissipate the repellent curtain at the tent entrance.On the other hand it is also quite possible that greater nepetalactone concentrations will be required to obtain effects that are comparable to the indoor performance of the system. Another important aspect that might have had a great impact on the outcome of the semi-field tests was the color and contrast conditions of the test site: while the BGS trap or a human volunteer was presented in front of a white background in laboratory trials, mosquitoes were exposed to a dark tent opening in semi-field tests (Figs 2 and 3). It has long been known that *Ae*. *aegypti* is highly attracted to dark colors [48; 49; 50] and the black interior of the tent could have diminished the impact of the push component.

A recent study investigated the spatial effect of catnip against stable flies in an outdoor situation [23]. Wax pellets containing 10% of the essential oil were dispersed in known stable fly resting areas and atmospheric concentrations of catnip oil volatiles were measured using solid-phase microextraction (SPME). Right at the start of the experiment approximately 160 ng/min of catnip oil volatiles were detected after a three minute exposure of the SPME fiber to the volatile laden headspace, correlating with a significant reduction in stable fly landing rates. The effect on the insects vanished after 24 hours and at this point in time the recovery of catnip oil volatiles had diminished to approximately 60 ng/min. Unfortunately, a cross-comparison of SPME and our purge & trap sampling method is not possible, but results of the cited study indicate that catnip oil can achieve spatial repellent effects against insects also in an outdoor setting, provided that a particular, critical threshold is reached. Future studies of the FFS should therefore include the evaluation of dispensers with higher catnip oil loading. In laboratory weighing experiments, catnip sachets lost around 0.06 g/h of catnip oil (representing approximately 13 mg of nepetalactone within a 15 min sampling period). Volatile sampling and TD-GC-MS analysis revealed nepetalactone quantities between 80–100 μg/m^3^ inside the air curtain generated by the FFS, which had an estimated volume of 0.25 m³. With an average wind speed of 0.9 m/s passing through an area of 0.14 m² in 15 min, a total air volume of 117 m³ was generated which took up between 9 to 12 mg of catnip oil volatiles (80–100 μg/m^3^ x 117 m³), thus quantification data correlate well with results from gravimetric mass loss measurements of the catnip dispensers. Future studies could investigate if dispensers filled with polymer granules holding higher doses of catnip oil have a greater spatial impact in semi-field trials. Likewise, using CO_2_ in an outdoor setting might boost BGS trap catch rates in the presence of a human volunteer and should be implemented in future studies.

## Supporting Information

S1 Fig Schematic drawing of the SHS (one unit). (TIFF)

S2 Fig Sketch of the air curtain generated by the SHS. Areas of lower density are indicated in grey. Air volume was estimated at 0.18 m^3^ (1.65 x 1.2 x 0.09 m)
(TIFF)

S3 Fig BGS recapture rates (means ± standard deviation) of *Ae*. *aegypti* in control trials of the FFS. The x-axis gives the different operating voltages with corresponding wind speeds, which were generated in the center of the tent opening (between positions 2 and 3 or at 77 to 107 cm above ground). Different letters indicate significant differences at p < 0.03 (Mann-Whitney-U-test, n = 10).
(TIFF)

S4 Fig Sketch of the air curtain generated by the FFS. Air volume was estimated at 0.24 m^3^ (1.7 x 1.2 x 0.12 m)
(TIFF)

S1 Table Wind speeds [m/s] within the FFS air curtain. Wind speeds were measured with an anemometer at 137 cm above ground (position 1), 107 cm (position 2), 77 cm (position 3) and 44 cm (position 4). Each position shows the minimum and maximum speed recorded in three individual measurements.
(PDF)
