# Correction: Evaluation of a push-pull approach for *Aedes aegypti* (L.) using a novel dispensing system for spatial repellents in the laboratory and in a semi-field environment

**DOI:** 10.1371/journal.pone.0134063

**Published:** 2015-07-24

**Authors:** Ulla Obermayr, Joachim Ruther, Ulrich R. Bernier, Andreas Rose, Martin Geier

There is information missing from funding section of this paper. Part of this research was funded by the European Union’s Framework 7 Health Innovation Initiative (agreement number: 306105, project acronym: MCD, http://www.mcdproject.org).
