# Supplementary figures and images for: Evaluation of a Push-Pull Approach for Aedes aegypti (L.) Using a Novel Dispensing System for Spatial Repellents in the Laboratory and in a Semi-Field Environment
Source: PLoS One. 2015 Jun 26;10(6):e0129878. doi: 10.1371/journal.pone.0129878 (PMC4482593; doi:10.1371/journal.pone.0129878)

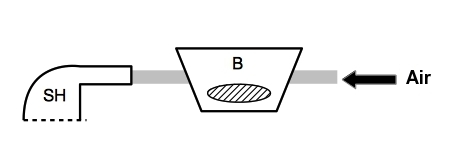

Supplement: S1 Fig — (TIFF) [file pone.0129878.s001.tiff]

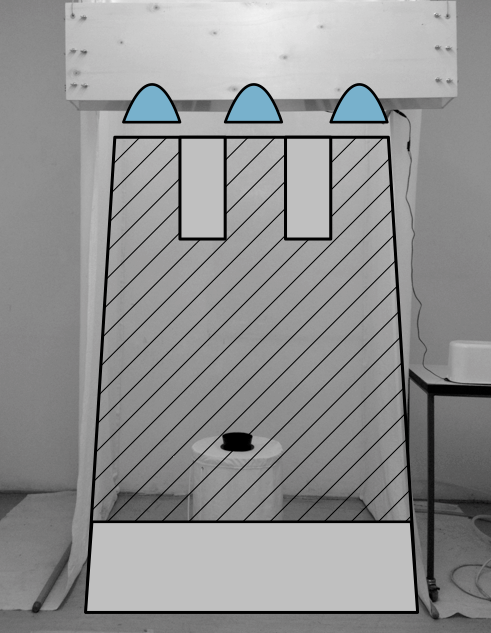

Supplement: S2 Fig — Areas of lower density are indicated in grey. Air volume was estimated at 0.18 m3 (1.65 x 1.2 x 0.09 m) (TIFF) [file pone.0129878.s002.tiff]

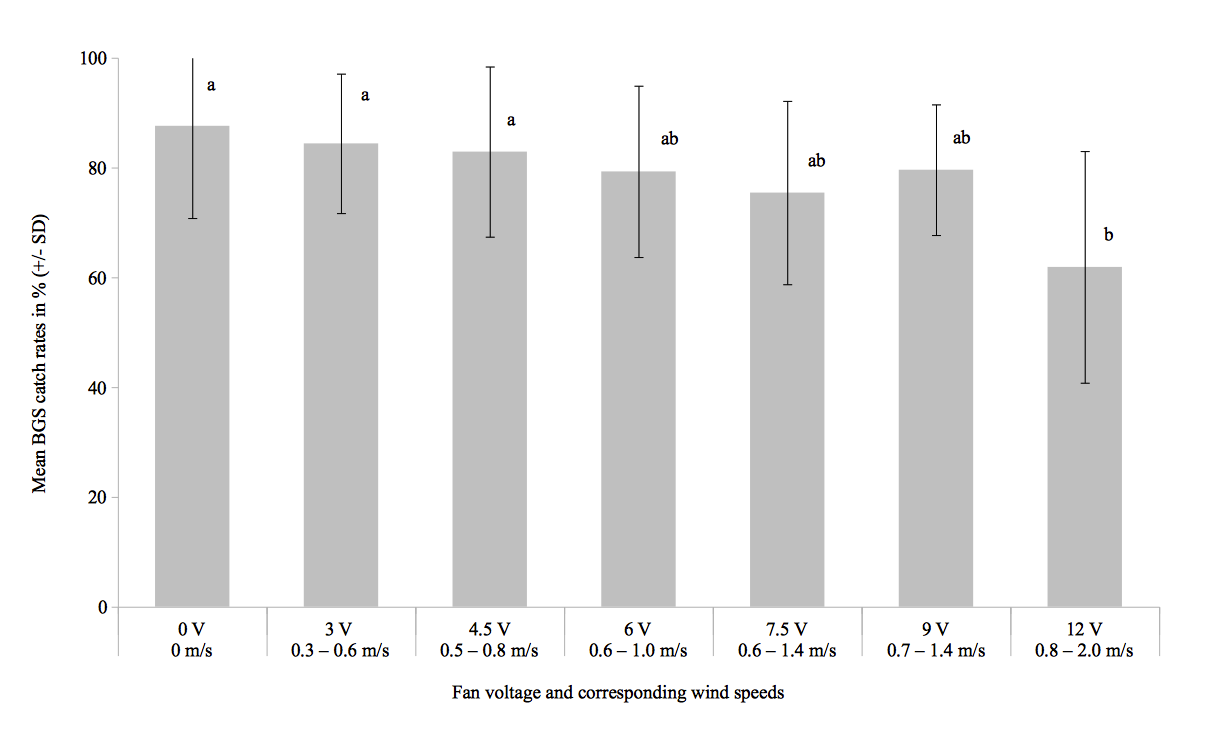

Supplement: S3 Fig — The x-axis gives the different operating voltages with corresponding wind speeds, which were generated in the center of the tent opening (between positions 2 and 3 or at 77 to 107 cm above ground). Different letters indicate significant differences at p < 0.03 (Mann-Whitney-U-test, n = 10). (TIFF) [file pone.0129878.s003.tiff]

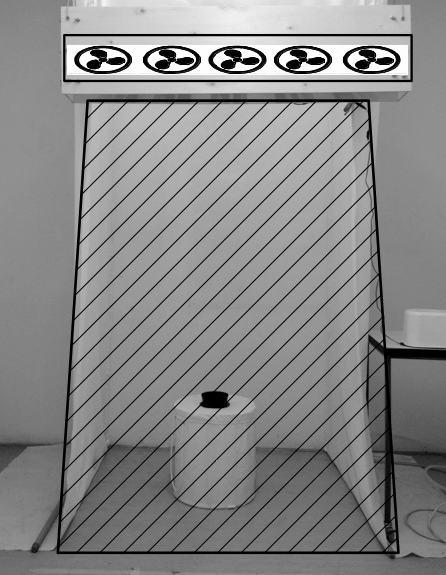

Supplement: S4 Fig — Air volume was estimated at 0.24 m3 (1.7 x 1.2 x 0.12 m) (TIFF) [file pone.0129878.s004.tiff]
