# Supplementary material for: Evaluation of a Push-Pull Approach for Aedes aegypti (L.) Using a Novel Dispensing System for Spatial Repellents in the Laboratory and in a Semi-Field Environment
Source: PLoS One. 2015 Jun 26;10(6):e0129878. doi: 10.1371/journal.pone.0129878 (PMC4482593; doi:10.1371/journal.pone.0129878)
Supplement: S1 Table — Wind speeds were measured with an anemometer at 137 cm above ground (position 1), 107 cm (position 2), 77 cm (position 3) and 44 cm (position 4). Each position shows the minimum and maximum speed recorded in three individual measurements. (PDF) [file pone.0129878.s005.pdf]

Position	3 V	4.5 V	6 V	7.5 V	9 V	12 V
1	0.7 – 1.0	0.8 – 1.5	0.8 – 1.4	0.9 – 2.0	0.9 – 2.0	1.0 – 2.4
2	0.4 – 0.6	0.6 – 0.8	0.6 – 1.0	0.9 – 1.4	0.9 – 1.4	0.8 – 2.0
3	0.3 – 0.5	0.5 – 0.8	0.6 – 0.8	0.6 – 0.7	0.7 – 0.8	0.8 – 1.0
4	0 – 0.1	0.1 – 0.2	0 – 0.2	0.1 – 0.2	0.1 – 0.4	0.2 – 0.4
